# Feasibility of an implementation intervention to increase attendance at diabetic retinopathy screening: protocol for a cluster randomised pilot trial

**DOI:** 10.1186/s40814-020-00608-y

**Published:** 2020-05-12

**Authors:** Fiona Riordan, Emmy Racine, Susan M. Smith, Aileen Murphy, John Browne, Patricia M. Kearney, Colin Bradley, Mark James, Mark Murphy, Sheena M. McHugh

**Affiliations:** 1 School of Public Health, University College Cork, Western Gateway Building, Western Rd, Cork, Ireland; 2 Department of General Practice, Royal College of Surgeons of Ireland, Dublin, Ireland; 3 Department of Economics, Cork University Business School, University College Cork, Cork, Ireland; 4 Department of General Practice, University College Cork, Cork, Ireland; 5 Medical Education Unit, University College Cork, Cork, Ireland

**Keywords:** Retinal screening, Family practitioner, Feasibility, Pilot trial, Implementation intervention

## Abstract

**Background:**

Diabetic retinopathy screening (DRS) leads to the earlier detection of retinopathy and treatment that can prevent or delay the development of diabetes-related blindness. However, uptake continues to be sub-optimal in many countries, including Ireland. Routine management of type 2 diabetes largely takes place in primary care. As such, there may be an opportunity in primary care to introduce interventions to improve DRS uptake. However, few studies test the feasibility of interventions to enhance DRS uptake in this context. Our aim is to investigate the feasibility of an implementation intervention (IDEAs (Improving Diabetes Eye screening Attendance)) delivered in general practice to improve the uptake of the national DRS programme, RetinaScreen.

**Methods:**

The IDEAs study is a cluster randomised pilot trial with an embedded process evaluation and economic evaluation. Following stratification by practice size, eight general practices (clusters) will be randomly allocated to intervention (*n* = 4) or wait-list control groups (*n* = 4). The intervention will be delivered for 6 months, after which, it will be administered to wait-list control practices. The intervention is multi-faceted and comprises provider-level components (training, audit and feedback, health care professional prompt, reimbursement) and patient-level components (GP-endorsed reminder with information leaflet delivered opportunistically face-to-face, and systematically by phone and letter). Patient inclusion criteria are type 1 or type 2 diabetes and DRS programme non-attendance. A multi-method approach will be used to determine screening uptake, evaluate the trial and study procedures and examine the acceptability and feasibility of the intervention from staff and patient perspectives. Quantitative and qualitative data will be collected on intervention uptake and delivery, research processes and outcomes. Data will be collected at the practice, health professional and patient level. A partial economic evaluation will be conducted to estimate the cost of delivering the implementation intervention in general practice. Formal continuation criteria will be used to determine whether IDEAs should progress to a definitive trial.

**Discussion:**

Findings will determine whether IDEAsis feasible and acceptable and will be used to refine the intervention and study procedures. A definitive trial will determine whether IDEAs is a cost-effective intervention to improve DRS uptake and reduce diabetes-related blindness.

**Trial registration:**

ClinicalTrials.gov NCT03901898. Registered 3rd April 2019,

## Background

Diabetes mellitus (diabetes) places a significant burden on health systems, largely attributable to the complications associated with the condition [1, 2]. The number of people with diabetes is growing worldwide, with type 2 diabetes accounting for approximately 90% of all cases [2]. Diabetic retinopathy (DR) is the most common microvascular complication of diabetes and a leading cause of blindness among people of working age in many countries [3, 4], including Ireland [5]. Worldwide, it is estimated that approximately 28 million individuals have vision-threatening retinopathy [6]. Visual impairment due to retinopathy can negatively impact on an individual’s quality of life and ability to self-manage which can lead to other complications [7].

Diabetic retinopathy screening (DRS) leads to earlier detection of pre-symptomatic retinopathy and treatment to prevent or delay the development of diabetes-related blindness. Screening for retinopathy is effective in reducing the risk of vision loss [8] and is cost-effective [9, 10]. National and international guidelines recommend annual DRS for people with diabetes [11–13]. Despite evidence for the effectiveness of DRS, uptake continues to be sub-optimal in many countries [14–17]. The current uptake of the national DRS programme in Ireland, in operation since 2015, is approximately 56% [18]. In England, where a national DRS programme has been established for > 10 years, attendance rates vary from 71–91% [19, 20]. Non-attendance for screening has been identified as a risk factor for poor visual outcomes among those with diabetes [21]. Non-attendance is also costly for the health service; retrospective analysis of 1 year of missed appointments within a primary care organisation in the UK was calculated to cost £78,259 [22].

A range of individual factors are consistently highlighted as being associated with DRS attendance; younger age [23, 24], social deprivation [24–27], longer duration of diabetes, type 1 diabetes [24], poorer glycaemic control, smoking and hypertension [28] and lack of awareness of DR and the risk [28, 29]. However, health care professional and system-level factors also play a part. For example, the accessibility of screening centres, time to attend DRS and competing demands have been identified as barriers [29]. On the other hand, communication and trust between health care professionals and patients, including a recommendation to attend screening from a primary care professional, is an important enabler [28, 29]. As most of the routine management of type 2 diabetes takes place in primary care, it is arguably the best setting for interventions to improve uptake of DRS.

An ‘implementation intervention’ is a type of intervention which supports *implementation* of a clinical programme, for example, DRS uptake; the intervention may be a multi-faceted approach comprising a number of different implementation strategies [30]. Interventions may be more successful if they are theory-informed, target known barriers and enablers of attendance [19, 31, 32] and operate at multiple levels (i.e., system, professional and patient) [33–36]. Interventions to improve screening attendance can be effective [37–39] and often include patient education to increase awareness of diabetic retinopathy and/or patient reminders, or registration and reminder systems to support professionals to follow-up patients [38]. Though these approaches can improve DRS uptake, few which target *both* professionals and patients [14, 40–44] focus on primary care [14]. There are challenges associated with introducing complex interventions in the primary care setting [45]. Interventions are not always a good fit for the context in which they are used or do not align with stakeholder preferences [46]. It is important, therefore, not only to develop a multi-level intervention informed by theory and guided by local stakeholder input [47], but to test whether it is feasible to deliver in the real-world primary care setting.

## Aims and objectives

The main aim of the IDEAs (Improving Diabetes Eye screening Attendance) study is to investigate the feasibility of a multifaceted implementation intervention in primary care to improve the uptake of the national DRS programme, RetinaScreen. The intervention was developed through a multi-stage process, combining theory, consultation with multiple stakeholders and existing evidence. In line with Medical Research Council guidance [31], the study will address uncertainties about feasibility, economic evaluation and the study procedures. Specifically, it will address the following questions:

1. Are the intervention content, delivery and procedures acceptable to people with diabetes who will receive it and staff who will deliver the intervention?
2. Is the intervention feasible to deliver in primary care practice and is fidelity achieved (i.e., intervention delivered as intended)?
3. Are the data collection processes, including mode and duration of data collection and outcome measures used, acceptable to staff?
4. Is the study feasible in terms of recruitment and retention procedures and data collection?
5. What are the costs associated with the intervention?
6. Do the results suggest that the intervention increases the uptake of DRS?

## Methods/design

### National screening programme

**Fig. 1:**
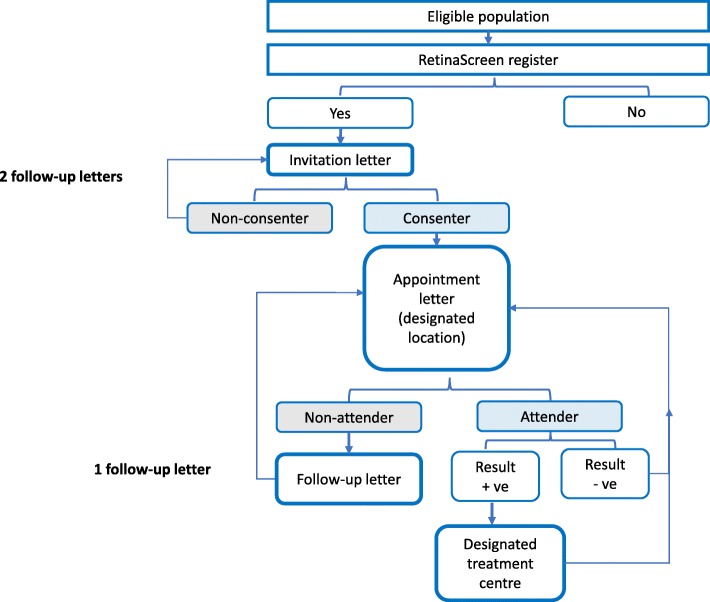
Flow diagram illustrating the process of consenting to and attending the programme [48]

In Ireland, a national DRS programme (RetinaScreen) was introduced in 2013 to offer free, regular diabetic retinopathy screening to people with diabetes. All people with diabetes who are older than 12 years old are invited by letter to participate in the programme [48], after which they provide consent for the programme to hold and use their contact details and receive an appointment. People have their screening appointment at one of the designated screening centres in a variety of community locations, including high street opticians, community health care centres and community hospitals. Some screening locations based within primary healthcare centres are co-located with other services, including general practice. In Ireland, GPs are not trained and funded to screen for DR. Following screening, participants who require further investigation and treatment are referred to one of seven treatment clinics nationally. The national programme works in conjunction with photography and grading providers (EMIS Care and Global Vision) to deliver the service*.* There is no national register of people with diabetes. The initial RetinaScreen register was populated in 2012 by using information from national health schemes. Those who were not captured by this method have to be added to the register by a GP or by other healthcare professionals involved in diabetes care. It is estimated that between 5.6 and 5.8% of the population of Ireland have diabetes, and this would equate to approximately 200,000 patients having the condition across Ireland. Based on figures reported by the national programme, as of December 2017, there were 164,569 men and women on the register, approx. 82% of the estimated population. Figure 1 illustrates the process of consenting and attending the programme.

### Study design

IDEAs is a cluster randomised pilot trial [49], with an embedded process evaluation and a partial economic evaluation (cost analysis). Eight general practices (clusters) will be randomly allocated to intervention (*n* = 4) or waitlist control (*n* = 4) groups following stratification by practice size. Practices in the wait-list control group will be offered access to the intervention material and support the following pilot trial completion at 6 months. Data from the trial will be analysed and reported in accordance with the CONSORT criteria. The results of the pilot trial will inform the design of a large cluster randomized trial that will be powered to test the effectiveness of the intervention on DRS uptake. Continuation criteria, based on quantitative and qualitative process evaluation data, will be used to inform the decision about whether the intervention should be further evaluated as part of a full-scale randomised controlled trial (Table 1).

**Table 1:** Summary of continuation criteria

^a^Continuation criteria	Measures used	Assessment of whether criteria have been met
Feasibility to recruit and retain practices.	• Number of practices responding to a call for Expression of Interest^b^ • Practice retention rates	If > 50 general practices within 2 months respond to call, then it is likely to proceed to full trial (*green*). If < 40 practices respond to call, then progression is unlikely to be feasible (*red*). If 40–50 practices respond within 2 month, then the Trial Steering Committee (TSC) will consider the feasibility of proceeding to the full-scale trial (*amber*) based on taking steps to improve response rate. If 8 practices are retained throughout a 6-month intervention period, then it is likely to proceed (*green*). If < 5 practices are retained, then a full-scale trial is unlikely to be feasible (*red*). If 5–7 practices are retained throughout the intervention period, then the TSC will consider feasibility of proceeding (*amber*) based on taking steps to improve retention.
Intervention is implemented as planned; that is, audit and feedback, addition of electronic prompts and delivery of a reminder in any format and receipt of intervention by eligible population of people with diabetes in participating practices	• Exploration of implementation fidelity during practice staff and patient interviews • Practice self-report during research support phone calls • Percentage of eligible patients receiving intervention based on practice audit data	If all core intervention components (audit, prompts, reminders) have been delivered by > 75% of practices, then it is likely to proceed to full trial (*green*) subject to review of qualitative data. If < 50% have delivered components, then it is unlikely to proceed (*red*). If 50–75% have delivered core components, then the TSC will consider the feasibility of proceeding (*amber*). The TSC will consider both the quantitative and qualitative data to judge whether the intervention is has been implemented.
Intervention is acceptable to patients and practice staff	• Percentages of staff reporting acceptability of intervention on self-report questionnaire. • Issues regarding acceptability of the intervention explored in qualitative interviews with staff and patients	If the intervention is acceptable to > 75% practice staff, then it is likely to proceed to full trial subject to review of qualitative data (*green*). If intervention is acceptable to < 50% staff, then it is unlikely to proceed (*red*). If the intervention is acceptable to 50–75% staff, then TSC will consider feasibility of proceeding (*amber*). The TSC will consider the quantitative and qualitative data and make an overall judgement on whether the intervention is acceptable.
Intervention has potential effect on screening uptake and demonstrates potential cost savings which are likely to outweigh the direct cost of implementing the intervention; specifically, the intervention (1) increases the absolute number of patients registered for diabetic retinopathy screening and (2) increases the absolute number of patients attending or intending to attend screening, measured descriptively	• Number registered for diabetic retinopathy screening based on practice audit data • Number attending or intending to attend based on practice audit data • Potential savings calculated based on cost of delivering intervention compared to control, and absolute increases in number attending or intending to attend	The TSC will judge whether the intervention has some positive impact and demonstrates cost savings.

^a^Continuation criteria are in place to facilitate the Trial Steering Committee (TSC) to assess whether it would be viable to progress to a full trial or whether this could be done following modifications to the trial protocol [50]. Continuation to the full trial will not occur unless problems can be overcome. Continuation criteria may be adapted, and supplementary data may be used to facilitate decision-making about whether to proceed to a full trial even when criteria have not been met

^b^To achieve 80% power to detect a change from 60 to 70% (*α* = 0.05), a total sample of 712 eligible patients would be needed. Assuming an average practice size of 1200 patients and a 5% prevalence of type 2 diabetes, at each practice, it is estimated that 12 patients would not be registered for the screening programme (20%). Of the 48 patients registered, 18 would be eligible (17 would be non-consenters (34%) and 1 would be a non-attender (3%)). Therefore, at least 40 practices would need to be recruited (20 per arm) for a full trial. To allow for a lower number of eligible patients per practice, we aim to recruit > 40 practices for the full trial

### Recruitment of general practices

Expressions of interest will be sought from general practices across Ireland through the Health Research Board (HRB) Primary Care Clinical Trials Network Ireland (CTNI) (*n* = 146 practices) and other networks known to the research team including; the Irish Practice Nurse Association (*n* = 1800 nurses), GP collaborators and discussion fora, national diabetes primary care initiatives (*n* = 79), the Irish Diabetes Nurse and Midwife Specialist Association, and social media posts. These networks are not mutually exclusive. Eligible practices will be required to have an electronic health record system and a practice nurse, to ensure they can deliver the intervention. Eight general practices will be purposively sampled from the list of interested eligible practices. As this is a pilot trial, a formal sample size calculation is not required [51]. A sample of eight was selected based on the resources available to conduct the pilot. The sample size is generally not a requirement of pilot studies [51]. The GP partner(s) will read an information sheet and provide consent on behalf of the practice.

#### People with diabetes

Inclusion criteria include the following:

- Aged 18 years or over
- Have diagnosed diabetes (type 1 or type 2)
- Are eligible to attend the national screening programme but have not attended the screening service (i.e., recently in the past 12 months or ever)

Individuals will be excluded if they have attended the DRS programme recently (i.e., in the last 12 months) or are known to be having retinopathy treatment.

### Allocating practices to trial groups

Given the investment of time and resources required to deliver the intervention, the number of clinical staff at the practice may influence the implementation and effectiveness of the intervention. Therefore, to achieve variations of experience, practices will be randomly allocated (using computer-generated random number (Excel system hosted in University College Cork)) to intervention or waitlist control groups in the ratio 1:1 following stratification by practice size (i.e., single/two-handed or group practice (≥ 3 GPs)). In order to reflect the distribution of GP practices nationally (52% with ≥ 3 GPs working) [52]. The invitation to take part in the study will be issued to the eight purposively selected practices by a member of the research team before randomisation sequencing. Due to pragmatic reasons, researchers will not be blinded to a group allocation of participating practices. Given the nature of the intervention, it will not be possible to blind participating practices.

### IDEAs intervention

**Fig. 2:**
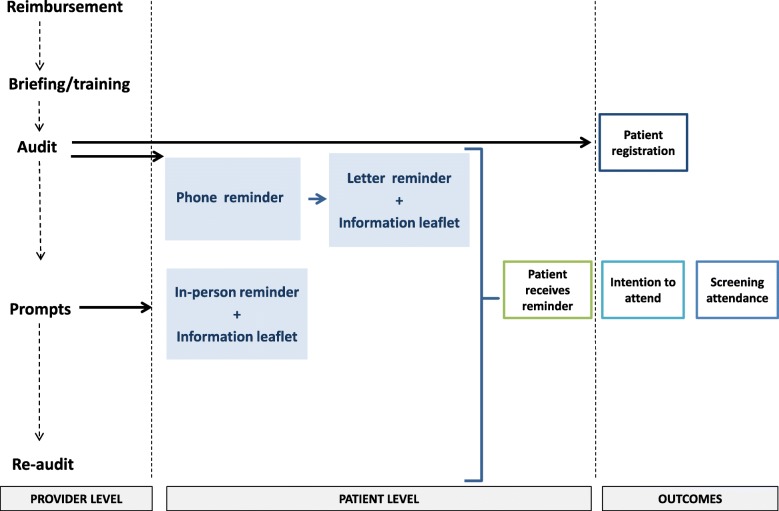
Overview of provider and patient level intervention components and study outcomes

The intervention will operate at the professional and provider level comprises training, audit and feedback, health care professional prompts, reimbursement for practices, and a GP-endorsed reminder for patients with information leaflet-delivered opportunistically face-to-face and systematically by phone and letter (Fig. 2). These components map to strategies proposed by Powell et al. [53] and support practice staff to deliver intervention components which target patients who have not attended screening previously (Table 2). The intervention incorporates a number of behaviour change techniques. The content of the intervention was developed using a theory-based four-stage process outlined by French et al. [54], drawing on qualitative research on the barriers and enablers of screening attendance (interviews with patients and health care professionals in Ireland) and an international systematic review of barriers and enablers of screening attendance [29, 54–56]. The acceptability and feasibility of intervention components were established through a consensus process with people living with diabetes and health professionals involved in diabetes care. Full details of the development of the intervention are in preparation for publication [57]. More details on the intervention content are provided in Additional file 1.

**Table 2:** Professional and patient level intervention components

Strategy according to ERIC^a^ [53]	Component
Audit and provide feedback	Practice audit of patients with diabetes (type 1 or type 2)
Conduct educational meeting	Briefing and training Briefing by the researcher for the practice team Training by researcher for the staff member responsible for conducting the audit
Provide local technical assistance	Supporting materials in the form of an audit and intervention manual
Remind clinicians	Electronic prompts Laminated script for face to face or phone encounters
Use other payment schemes	Reimbursement to practices
Intervene with patients	Reminder messages delivered face-to-face or via phone GP-endorsed reminder letter and information leaflet

^a^Experts’ recommendations for implementing change project

#### Audit and provide feedback

A staff member at each participating practice (administrator or practice nurse) will be assigned within the practice to conduct an audit of their patients with diabetes (type 1 or type 2) aged ≥ 18 years. This audit is necessary as RetinaScreen does not provide registration and attendance data at the practice level. Patient’s individual records will be checked by the practice staff member for a ‘results’ letter from the national screening programme to identify all people who have not attended the screening with the national programme. Patients may be classified as follows:

- ‘Attenders’, patients who have any letter from RetinaScreen, i.e., the results’ letter indicating previous attendance at some time
- ‘Recent non-attender’, i.e., they may have a results letter(s) for older appointment(s) (more than 12 months ago) but have either a Did Not Attend (DNA) letter for their last appointment or no letter in the past 12 months
- ‘Non-attender’, i.e., DNA letter(s) and/or no results letter(s)
- ‘No record’, i.e., no record on file; status to be verified

If there is no evidence (e.g., letter) on the patient file to indicate they are registered with the national screening programme, the staff member will verify their status through the dedicated national telephone line or online portal. Practices will be asked to complete the first step of the audit within a maximum of one calendar month. Practices with large numbers of people with diabetes may not have the capacity to audit all their patients. Therefore, practices which have > 100 patients with diabetes will be asked to audit a random sub-sample of 100 patients. They will receive advice and support from the research team on this process. They will be asked to record the total number of patients with diabetes and retain a file with some basic demographic information (age, gender, type of diabetes) to examine whether the audited sub-sample is different from patients who were not sampled. The baseline audit will be completed within one calendar month of starting the trial. At 6 months, practices will re-audit eligible patients. As the 6-month period is insufficient for all patients to attend and receive a confirmation letter from RetinaScreen, practices will also verify their status via phone call, i.e., to determine whether they (a) intend to contact RetinaScreen, (b) have contacted RetinaScreen or (c) have attended the screening. If they do not fulfil (a), (b) or (c), then, they will be asked why, and this information is recorded on the audit file. Audit data will be made available to study researchers after it has been fully anonymised at the practice.

#### Educational meeting and provision of local technical assistance

Before the audit of patients with diabetes, the staff member responsible for conducting the audit will receive one-on-one training from a study researcher. Technical assistance with the audit will be provided by the researcher in the form of an audit manual. Training duration is estimated to take 1 h but is expected to vary according to practice experience conducting an audit. This training will be preceded by a brief, 20–30 min session on the intervention and its delivery. This session should be delivered to the entire practice team if possible. The researcher will outline the estimated time each intervention component is expected to take. For example, the audit has been piloted in a large primary care centre as part of previous work by the research team and is estimated to take on average 5 min per patient. GP collaborators on the research team have conducted a pilot (e.g., 2–3 patients) using the audit protocol to check clarity, usability and time required.

#### Remind clinicians

After the audit has been completed, feedback (i.e., a list of patients who have not attended screening) will be used by an assigned staff member to add electronic delivery prompts to the records of eligible patients. This will act as a reminder to prompt professionals (GPs or practice nurses) to deliver the face-to-face reminder message to patients and to record delivery of this message. Health care professionals will be asked to delete the alert if the intervention components (i.e., brief messages, leaflets) were delivered where the functionality to capture action or inaction on the alert exists in the GP software this will be utilised.

#### Payment scheme

Practices will be reimbursed for the cost of taking part in training, conducting the audit and delivering the intervention, including any consumables. Practices will be paid a fee (a maximum of €1000 per practice) based on estimated salary costs, time to deliver the intervention and allowances for overheads. Practices will be reimbursed at the start of the study (€500) and upon receipt of the audit file at study completion (maximum of €500) by the research team.

#### Intervene with patients to enhance uptake and adherence

The patient-level intervention consists of reminders delivered opportunistically face-to-face, and systematically by phone and letter accompanied by an information leaflet. These components will be delivered after the audit has been completed and prompts added to electronic records.

##### Verbal reminders

All eligible patients will receive a phone call from a practice nurse, reminding them to attend the screening. Nurses who deliver the phone call will follow a short script (Additional file 2) and inform the patient they will receive a follow-up letter with more information. At this point, the patient status will also be updated on the audit file and electronic patient record, for example, if the patient is attending another service for screening (e.g., ophthalmologist). This will enable staff to shortlist patients who should receive the letter. In-person reminders will be delivered opportunistically by GPs and practice nurses if an eligible patient attends for an appointment during the 6-month trial period. They will follow the short script (Additional file 2) and provide patients with a standard information leaflet (Additional file 3).

##### GP-endorsed letter and information leaflet

Following the phone call, patients will receive a reminder letter, recommending participation in the national retinopathy screening programme, together with key messages (Additional file 2). A copy of the consent and registration form developed by RetinaScreen, a freepost envelope, and the information leaflet will be included with the letter.

Reminders will be issued to patients during a 2-week period following the audit. During this time period, it will be specified that the practice should make a reasonable attempt (i.e., three attempted phone calls) to remind patients using the approaches outlined, after which the letter should be issued.

All materials have been designed to be understood by readers with low literacy, numeracy or both, with input from healthcare providers and patients. Materials have been reviewed by the National Adult Literacy Agency and the IDEAs Patient and Public Involvement (PPI) group.

### Control practices

In control practices, data collection will be carried out at 6 months, using date-restricted data extraction from the electronic medical record to capture data for the full 12-month period prior to the intervention, so this includes baseline and 6 months of follow-up data. This will match the 6-month period in intervention practices, during which they will have acted as control practices. This will ensure we have comparative data collection prior to the delivery of the intervention to the control group on study completion. This approach was chosen as collecting data at baseline (i.e., 6 months before study start) would constitute an intervention in those practices; knowledge of non-attenders would lead to a change in usual care as the control group would likely follow-up patients immediately. Control practices will receive the same supports and training as intervention practices to facilitate the audit feedback loop.

Implementing the patient-level intervention will not require alteration to usual diabetes care (including the use of any medication). Usual care will continue for both trial arms.

### Outcomes

Primary outcome measures will be the following:

1. Patient registration
2. Patient response to reminders: (a) *intention* to contact RetinaScreen, (b) have contacted RetinaScreen, (c) have attended a screening or (d) none of these. This will be verified through a phone call from the practice as part of the 6-month audit.
3. Patient attendance at retinopathy screening, verified through a letter received by practices from the national screening programme, RetinaScreen. Patient attendance will be measured (via practice audit) at baseline and at 6 months.

**Fig. 3:**
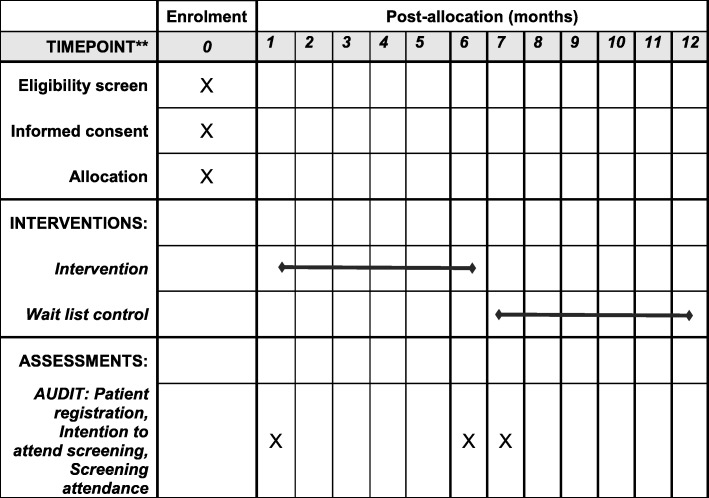
Schedule of enrolment, interventions and assessment

Figure 3 shows the schedule of enrolment, interventions and assessments.

### Sample size

As this is a pilot trial, formal sample size calculation has not been carried out [51]. The purpose of the pilot is to investigate the feasibility and acceptability of the intervention and study procedures with a view to adapting these, if necessary, for a future definitive trial. Eight practices will be recruited. Based on previous work conducted by the research team, within a practice, it is expected that 20% of patients will not be registered. Of those who are registered, it is expected 34% will not have consented to the programme, and 3% will not have attended. Therefore, assuming an average practice size of 1200 patients and a 5% prevalence of type 2 diabetes, at each practice, it is estimated that 12 patients would not be registered for the screening programme (20%). Of the 48 patients registered, 18 would be eligible (17 would be non-consenters (34%) and 1 would be a non-attender (3%)).

### Data collection

As part of the audit, in addition to screening status and *intention* to attend the screening as described above, the following data will be collected: age, gender, diabetes type, general medical service status, private health insurance, duration of diabetes and treatment type (diet, oral agents, injectables, insulin).

## Process evaluation

### Recruitment

#### General practice staff

All staff at selected practices (GPs, practices nurses and administrators) will be eligible to participate in the process evaluation. A purposive sample of staff who are participating in the study will be contacted by a study researcher following study completion and invited to participate in qualitative interviews.

#### People with diabetes

At study completion, practices will send a letter to, or phone, all eligible patients informing them that the research team is working with their general practice to evaluate a practice approach to promoting screening. Patients who are interested in taking part can provide their contact details to the study team. A member of the research team will then invite them to an interview or focus group and ask them two screening questions (i.e., age and if/when they last attended) to facilitate purposive sampling of participants.

### Data collection

In line with the Medical Research Council guidance [31], a mixed methods approach will be used, involving data collection at the practice, professional and patient level, to assess implementation outcomes, as defined by Procter et al. [58], namely appropriateness, acceptability, feasibility, reach/penetration, fidelity and costs (resource use). A summary is provided below (Table 3) with more detail and definitions available in Table 4.

**Table 3:** Summary of data collection

Practice level	
• **Audit;** ongoing data collection (i.e., number of eligible patients, number of reminders delivered) [reach, fidelity]	
• **Research processes;** data collection at study start (i.e., recruitment and retention, briefing attendance and feedback), and via monthly phone calls to practices (i.e., role assignment, changes to the intervention protocol, time and resource use, any feedback) [feasibility, fidelity, costs (resource use)]. Information collected will vary according to timing of the phone call. For example, during the first phone call, the time taken to conduct the audit will be recorded; during the second call, the time needed to deliver reminder phone calls will be recorded***.***	
Professional	
• **Staff questionnaires** at study cessation [appropriateness, acceptability and feasibility]	
• **Staff interviews** at study cessation [acceptability, feasibility and fidelity]	
Patient	
• **Patient interviews or focus groups**, at study cessation [acceptability and fidelity]

**Table 4:** Data collection conducted as part of the process evaluation

**Data items**	**Timing**	**Outcome(s)**					
		**Appropriateness**^**b**^	**Acceptability**^**b**^	**Reach**^**b**^	**Feasibility**^**b**^	**Fidelity**^**b**^	**Costs**^**a**^
**Source:** Audit data **Level:** Patient **Content:**				✓		✓	
• Number of patients eligible to receive the intervention	Baseline						
• Number of phone reminders; record on the audit file that (a) patient phoned (and date), (b) spoke to the patient and (c) delivered the scripted message.	On-going						
• Number of letter reminders	On-going						
• Number of in person reminders; review patient files and record whether the electronic alert has been deleted. Deleting the electronic alert will be taken to indicate that the intervention was delivered (self-report).	At 6 months						
**Source:** Research processes **Level:** Practice **Content:**					✓	✓	✓
Recruitment • Recruitment and retention rates, which will be used to inform the sample size calculation for the definitive trial • The time taken to recruit practices, collect data and install the intervention (i.e., training and audit set up) to inform the planning of a larger definitive trial	At recruitment Baseline				✓		
Feedback • Any complaints or feedback about the intervention or research process during the trial	During delivery				✓		
Briefing • Number and type of staff who attend (i.e., practice nurse, GP, administrator); • Time taken to deliver briefing/training; • Participants who attend will be asked for suggestions on how to improve the briefing; • Usual care at the practice, that is, whether they already remind patients, and how this is done at the practice.	During delivery		✓		✓	✓	✓
Researcher phone calls • Staff member assigned to delivery of different intervention components • Why they were assigned to this role • Changes to role assignment and reason for change • Changes to intervention protocol • Any additional resources used to deliver the intervention; for example, additional appointments resulting from the phone call or letter, further phone calls as a follow-up to the original reminder (e.g., ringing the patient back to confirm some detail) • Number of patients who contacted practice for information or came to the practice looking for help with the consent form. • Estimate of the time commitment to the study including time taken to participate in audit and feedback (including verification with programme), incorporate face to face reminders into consultations and to deliver reminder phone calls and issue letters.	Monthly, and reviewed at 6 months				✓	✓	✓
**Method:** Staff questionnaires **Level:** Practice **Content:** • Acceptability of Intervention Measure (AIM) • Intervention Appropriateness Measure (IAM) • Feasibility of Intervention Measure (FIM)	At 6 months	✓	✓		✓		
**Method:** Staff interviews **Level:** Practice	At 6 months		✓		✓	✓	
**Method:** Patient interviews or focus groups **Level:** Patient	At 6 months		✓			✓	

^a^Data collected on resource use, after which monetary values will be assigned to give cost estimates as part of the economic evaluation

^**b**^According to Proctor et al., appropriateness is the ‘perceived fit, relevance or compatibility of the innovation or evidence-based practice for a given practice setting, provider, or consumer and/or perceived fit of the innovation to address a particular issue or problem’**;** Acceptability is the ‘perception among implementation stakeholders that a given treatment, service, practice or innovation is agreeable, palatable or satisfactory’**;** Reach (penetration) is defined as the ‘integration of a practice within a service setting and its subsystems’; Feasibility is defined as the ‘extent to which a new treatment, or an innovation, can be successfully used or carried out within a given agency or setting’ [58]

#### Audit data and research processes

The staff member responsible for the audit will record the number of patients eligible to receive the intervention and the number of reminders delivered. The researcher who delivers the briefing and training will record information on attendees, time required, usual care at the practice and ask for suggestions on the delivery and content of the briefing. Information from monthly phone calls will be recorded on a standardised extraction form.

#### Staff questionnaires

At study completion (6 months), staff at participating practices will be asked to complete a questionnaire containing three previously validated measures, to assess the acceptability, appropriateness and feasibility of the intervention [59]. Acceptability is defined the ‘perception among implementation stakeholders that a given treatment, service, practice or innovation is agreeable, palatable or satisfactory.’ Appropriateness is the ‘perceived fit, relevance or compatibility of the innovation or evidence-based practice for a given practice setting, provider or consumer and/or perceived fit of the innovation to address a particular issue or problem’. Feasibility is defined as ‘the extent to which a new treatment, or an innovation, can be successfully used or carried out within a given agency or setting’ [58, 59]. Practice staff will also be able to provide additional comments on the intervention, specifically the fidelity, acceptability, suitability and/or comprehensiveness of the study and intervention procedures, and how these might be improved.

#### Staff interviews

A purposive sample of staff (GPs, practice nurse, practice manager, administrator) will be invited to participate in semi-structured interviews. The initial sample will comprise 12 staff members (one GP, practice nurse and administrator or manager from each of four intervention practices). Drawing on the principles of theoretical saturation and data saturation [60–62], data will be analysed at this point. Saturation will be judged at the practice level. Therefore, further sampling may be directed to pursue new topics arising which are specific to one practice. Where numbers allow, three further interviews with different staff members (one GP, nurse and administrator/manager) will be conducted to determine whether new topics within the main implementation constructs, acceptability and feasibility, arise. If necessary, further interviews will be conducted in blocks of three.

Topic guides will be informed by existing implementation frameworks to explore key constructs, including but not limited to the Theoretical Framework of Acceptability [63], Consolidated Framework for Implementation Research [64] and the Framework for Reporting Adaptations and Modifications-Enhanced (FRAME) [65, 66]. Interviews will explore staff members’ perspectives and experiences of the intervention and research procedures, fidelity to the intervention process (delivered as planned or whether adaptations to the intervention were made during delivery) and the overall feasibility and acceptability of the study procedures and the intervention, including strengths and weaknesses of the intervention, and key challenges (barriers and facilitators) to implementation in practice. Beliefs about the capacity and resource need to deliver the intervention, training needs and contextual influences on implementation will be explored. Data collection and analysis will be iterative. A separate consent will be obtained from staff who participate in the end of study interviews.

#### Patient interviews or focus groups

Interviews will also be carried out with patients with type 1 or type 2 diabetes who received the patient-level intervention. A purposive sampling strategy will be used to recruit participants from intervention practices (2–3 patients from each practice) on the basis of age, sex and attendance pattern (i.e., never attender, recent attender). A semi-structured topic guide will be used to elicit feedback on their experiences and perspectives of the intervention, whether the intervention was acceptable, advantages and disadvantages, and the perceived influence on behaviour. The topic guide will be informed by previous literature on barriers to screening attendance [29], and the theoretical basis for how the intervention is expected to work. If it is unfeasible to arrange focus groups, or if patients are unable to attend, then they will be given the option to take part in an interview. At the start, patients will be asked to complete a short 6-item questionnaire, asking them their age, how long they have diabetes, the type of diabetes, their occupation, whether someone in their family has diabetes and if they have any existing diabetes-related complications either now or in the past. Patients will not be obliged to complete the questionnaire to participate in the focus group or interview.

#### Consent

Study researchers will provide eligible staff and patients with information leaflets and consent forms. Staff and patients will sign a consent to participate in interviews or focus groups. Staff will complete a separate consent form to complete questionnaires at study cessation.

#### Economic evaluation

The economic component of the study will consist of a cost analysis of the trial and budget impact analysis of the strategy. Concurrent with the pilot trial, data collected will be employed in a cost-analysis to establish the cost of delivering the strategy compared to the control (calculated as per national guidelines [67]). Appropriate one-way sensitivity analyses will be conducted around key parameters to investigate parameter uncertainty. In addition, a budget impact analysis (BIA) will be conducted in line with national guidelines [68] to predict the potential financial impact of the adoption and diffusion of the intervention in the short term (up to 5 years as per HIQA guidelines). The results of these economic analyses used to inform decisions regarding resource or budget planning for a definitive trial.

### Analysis

#### Quantitative analysis

As this is a pilot trial, the analyses will focus on describing the key process measures to decide if the main trial is feasible and desirable. Descriptive analyses will be conducted for the primary outcomes; however, this will be treated as exploratory. Using anonymised audit data, a baseline table (descriptive statistics and frequencies) will compare the demographic and clinical characteristics of the intervention and control groups. Descriptive summaries will be generated for the patient outcome, screening intention and attendance. The data will provide information on the parameters for an accurate sample size calculation (mean, standard deviation and intervention effect) for the future definitive trial. Descriptive analysis will be conducted to assess practice recruitment and retention rates, and reach. All data will be managed and analysed using Stata V14 software.

#### Qualitative analysis

All focus groups and interviews will be digitally recorded and transcribed verbatim. Transcripts will be entered into the NVivo qualitative analysis software to facilitate data management, coding and retrieval. Transcripts will be coded using the Framework Method [69], drawing on existing frameworks. For example, FRAME will be used to code modifications to the intervention; specifically, the *process* of modification (what was modified, at what level, who decided to make the modification, the nature of the modification, when it occurred, whether it was planned and whether it was fidelity-consistent or inconsistent) and the *reason(s)* (socio-political, organisational, provider, recipient). The framework approach is suitable for projects with prespecified objectives such as evaluating acceptability and feasibility. It also allows researchers to develop unexpected themes during initial phases of familiarisation and open coding and facilitates cross-group comparison allowing us to examine themes among different types of practices and patients.

#### Integration

Where available, quantitative and qualitative data will be integrated to ensure a comprehensive, multi-perspective approach to exploring the intervention process. Table 5 outlines which data will be integrated for different constructs. For example, findings from the qualitative analysis will be integrated with quantitative data to assess fidelity, feasibility and acceptability [70]. Quantitative and qualitative data collected on intervention delivery will be classified according to existing frameworks for coding adaptation and modifications to interventions [65, 66].

**Table 5:** Summary of data integration

Research question	Implementation outcome	Data
**Intervention**		
1. Are the intervention content, delivery and procedures acceptable to people with diabetes who will receive it, and staff who will deliver the intervention?	Acceptability	**Quantitative:** staff questionnaires **Qualitative:** staff and patient interviews or focus groups; any feedback on intervention content during the study
2. Is the intervention feasible to deliver in primary care practice and is fidelity achieved?	Feasibility	**Quantitative:** staff questionnaires, number and type of staff who attend briefing, role assignment, time and resource use **Qualitative:** staff interviews, any feedback on intervention during the study
	Fidelity & adaptations	**Quantitative:** number of patients eligible to receive the intervention and number of reminders delivered, number and type of staff who attend briefing
		**Qualitative:** staff and patient interviews and focus groups data on any changes to role in intervention delivery and the reason for the change, any changes to the intervention protocol.
3. What are the costs associated with the intervention?	Costs	**Quantitative:** number and type of staff who attend briefing, role assignment, time and resource use
**Study procedures**		
4. Are the data collection processes, including mode and duration of data collection and outcome measures used, acceptable to staff?	Acceptability	**Qualitative:** staff interviews, any feedback on research processes during the study
5. Is the study feasible in terms of recruitment and retention procedures and data collection?	Feasibility	**Quantitative:** recruitment (level of interest) and retention rates, time taken to set up practices, conduct practice visits, deliver briefing/training and monthly phone calls **Qualitative:** any feedback on research processes during the study

As this is a pilot study, the level of missing data will be documented, but no imputation will be undertaken. As outlined, adherence to the study protocol (fidelity assessment) will be assessed throughout the trial period and at trial completion as part of the process evaluation. Exploratory descriptive analysis of the primary outcome will be conducted using the intention-to-treat approach.

### Trial oversight

The sponsor of the trial is the University College Cork. Day-to-day management of the trial is the responsibility of the PI. The TMG comprising the PI (SMH) and grant co-applicants, JB, SMS, PMK and AM has been set up to assist with this function. Independent overall supervision of the trial is provided by the Trial Steering Committee (TSC) which is composed of an independent Chairperson, two expert independent members, one Principal Investigator (PI), one lay representative and two representatives from the TMG (non-independent members). The TSC will meet four times over the course of the trial. The TSC will report their decisions in writing to the TMG, within 1 week of the meeting where possible. It is the responsibility of the TMG to implement any actions required. It is the responsibility of the chair to report these decisions. A Data Monitoring Committee (DMC) is not required for this trial due to low-risk nature of the trial, the lack of interim data and the short-term follow-up of 6 months.

### Data management

All data will be collected, used, stored and otherwise processed in accordance with the Data Protection Acts (DPA) 1998–2018. Patient data will be anonymised within the practice before being shared with the research team, collated on an encrypted laptop and transferred to the university campus. All anonymised files will be merged into a single study database for checking, cleaning and analysis in Stata statistical software. Anonymised data will be stored electronically on the secure UCC server and will be password protected. Personal patient data (all audit files) will only be retained in the practice and will be only accessible to staff who otherwise have access to this data to deliver patient care. All participants’ personal identifiable data (PID) collected as part of the process evaluation will be stored electronically on the secure UCC server and will be password protected. A unique ID number will be assigned to each participant. The key linking participants to ID numbers will be stored separately and securely, and only the named researchers will have access to the data and the key. Given that consent forms may also potentially identify participants, these will be stored securely and separately from the ID key in a locked filing cabinet. Only the investigators named on the application approved by the Research Ethics Committee will have access to the data collected as part of the study.

### Monitoring

Due to the nature of the intervention, no serious adverse events or adverse events are anticipated. However, to capture any complications associated with the trial, participating practices will be asked during monthly phone calls whether they have any complaints or feedback about the intervention or research process. There are no formal stopping rules; however, details of any issues or adverse events reported by participating practices will be considered by the Trial Management Group (TMG). Interim analyses will not be conducted due to the low-risk nature of the trial, the lack of interim data and the short-term follow-up of 6 months.

### Dissemination

The results from this study will be published in a peer-reviewed journal for dissemination amongst researchers and clinicians. We will follow the International Committee of Medical Journal Editors (ICMJE) recommendations for authorship and review these for each individual publication. Presentations of study findings will also be taken to relevant national and international research conferences. The results will also be disseminated to participants, if they agree to this. Important protocol modifications will be reported to trial registries, the Research Ethics Committee and journals.

### Patient and public involvement

An IDEAs PPI group, comprising of five people with diabetes has been established; three women and two men, two with type 1 diabetes. The PPI group has advised on the development of the study materials, specifically the wording of the GP-endorsed letter and standardised script, and the format and content of the patient information leaflet. The group will continue to be involved throughout the study. A lay member has been recruited to sit on the TSC.

## Discussion

The aim of this cluster randomised pilot trial and process evaluation is to examine the acceptability and feasibility of an intervention to improve DRS uptake. Internationally, attendance at screening continues to be poor [14–17]. With a greater proportion of management of type 2 diabetes taking place in the community [71], professionals in primary care are well placed to promote screening attendance among people with diabetes. Few studies examine ways to target *both* professionals and patients [14, 40–44], to promote screening uptake in this setting [14]. Given the importance of trust and communication between health care professionals and patients [28, 29], interventions to improve uptake should include components which support professionals to endorse screening in a targeted way, i.e., among people who do not attend.

The current intervention was developed through a systematic multi-stage process combining theory, evidence and consultation with multiple stakeholders. Components found to improve uptake of retinopathy screening [38, 43, 72] and cancer screening [73–82], include audit and feedback [83], patient [73, 76, 77, 79, 84–91] and physician [38, 72] reminders, the use of trusted sources to deliver messages [37, 73–75] and key information leaflets [74, 81, 82]. Our intervention comprises these strategies. As mentioned, successful delivery of the intervention in general practice may be affected by several factors, including workforce shortages [92–95], workload and time constraints [96], and other demands on the service. Furthermore, the recent introduction of remuneration for GPs in Ireland to provide structured care to certain patients with diabetes (i.e., only those holding a means-tested general medical services (GMS) card; a public insurance scheme which entitles cardholders to free access to their GP) [20] could mean patient groups are managed differently in general practice according to their healthcare cover. We acknowledge this intervention does not address language barriers in population subgroups and may need to be adapted to improve cultural and linguistic ‘fit’ with some populations [57]. As part of the process evaluation, we will examine fidelity and adaptations and whether patients considered the intervention appropriate for them. Another relevant context for the current study is the recent National Cervical Screening Programme controversy, whereby women were not informed of inaccurate smear test results [97]. There are concerns that this controversy has undermined public confidence in screening and may have implications for the uptake of other programmes run by the National Screening Service, including DRS.

The strength of this pilot trial is that it will be conducted in practices across Ireland, which differ in geographic location and size, enabling the feasibility and acceptability of the intervention in different contexts to be explored [98]. While the core intervention components have been established, there is some flexibility to facilitate its ‘fit’ with existing practice resources. Although we will advise practices on staff members who are best placed to deliver certain components, practices will be able to assign staff as they deem appropriate. One of our objectives will be to explore in detail any adaptations to the intervention content and delivery, and why these occurred. A further strength is the inclusion of an economic evaluation, which often does not form a standard part of the assessment of implementation research [99]. Understanding the costs and benefits associated with interventions is crucial to decide whether and how they can potentially be delivered as part of everyday practice. Study findings will determine whether it is feasible to conduct a full economic evaluation of the IDEAs intervention, specifically, that is viable and feasible to capture resource use and outcome data as part of a full-scale trial.

One limitation is the potential for selection bias. Practices who are interested in improving patient attendance and feel they have the capacity at the practice to do so are more likely to respond to the call for expressions of interest. Many recruitment avenues utilised by the study team are likely to be populated by professionals with an existing interest in research and quality improvement, who may have better systems and processes in place, making it more feasible for them to deliver the IDEAs intervention. A further limitation is that information on research processes provided by practice staff (as part of monthly phone calls), and fidelity as relayed during interviews at study completion, could be subject to recall and self-report biases. Electronic alerts may be deleted by professionals regardless of whether they have delivered the in-person reminder. It is not within the scope of the study to modify practice software to capture action or inaction on the alert, unless this functionality already exists. Measuring resource use for the economic evaluation relies on the accuracy of staff recall, specifically the time taken to deliver components, and whether additional resources were used. However, an observational time-in-motion study would be inappropriate given confidentially concerns and the potential to breach the General Data Protection Regulations (GDPR).

This study is the first to examine the feasibility of an implementation intervention to enhance the uptake of DRS in Ireland, and one of few studies examines interventions targeting *both* professionals and patients which promote screening uptake in primary care. Results will be used to improve the intervention and study procedures with a view to progressing to a definitive trial. This will ultimately determine whether IDEAs is an effective and cost-effective intervention to improve DRS attendance. Although DRS meets the World Health Organization criteria for a screening programme [100], few countries have introduced a national population-based DRS programme. When establishing and embedding a national screening programme, it is important that effective interventions to drive attendance are employed from the outset. The current intervention is an exemplar of how to connect local health services (i.e., general practice) to a national population-level programme to support implementation. The long-term aim is to ultimately reduce DR-related sight loss, through encouraging people to participate in the national programme, facilitating early detection of DR and access to appropriate treatment.

## Supplementary information

**Additional file 1:.** The TIDieR (Template for Intervention Description and Replication) Checklist

**Additional file 2:.** Messages delivered as part of the short script. Intervention material

**Additional file 3:.** Information leaflet and intervention material

## Data Availability

Data sharing is not applicable to this article as no datasets were generated or analysed during the current study.

## Abbreviations

CTNI: Primary Care Clinical Trials Network Ireland
DMC: Data Monitoring Committee
DNA: Did Not Attend
DR: Diabetic retinopathy
DRS: Diabetic retinopathy screening
FRAME: Framework for Reporting Adaptations and Modifications-Enhanced
GP: General practitioner
HRB: Health Research Board
IDEAs: Improving Diabetes Eye screening Attendance
PPI: Patient and Public Involvement
TMG: Trial Management Group
TSC: Trial Steering Committee
