# Supplementary material for: Feasibility of an implementation intervention to increase attendance at diabetic retinopathy screening: protocol for a cluster randomised pilot trial
Source: Pilot Feasibility Stud. 2020 May 12;6:64. doi: 10.1186/s40814-020-00608-y (PMC7216495; doi:10.1186/s40814-020-00608-y)
Supplement: Supplementary file 1 — Additional file 1:. The TIDieR (Template for Intervention Description and Replication) Checklist [file 40814_2020_608_MOESM1_ESM.docx]

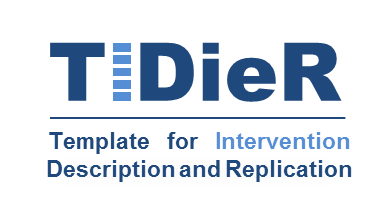


**The TIDieR (Template for Intervention Description and Replication) Checklist**

Information to include when describing an intervention and the location of the information

| **Item number** | **Item** |  |
| --- | --- | --- |
|  |  |  |
|  | **BRIEF NAME** |  |
| **1.** | Provide the name or a phrase that describes the intervention. | Improving Diabetes Eye-screening Attendance (IDEAs) intervention |
|  | **WHY** |  |
| **2.** | Describe any rationale, theory, or goal of the elements essential to the intervention. | The intervention was developed using the process described by French et al. and aims to improve uptake of diabetic retinopathy screening.  The following Behaviour Change Techniques are incorporated in the patient-level intervention: 1. Prompt/cue; 2. Information from a credible source; 3. Information on other’s approval; 4. Information on health consequences; 5. Framing/reframing; 6. Reduce negative emotions; 7. Verbal persuasion to boost self-efficacy; 8. Information on social and environmental consequences; 9. Instruction on how to perform the behaviour; 10. Social support (practical*)* |
|  | **WHAT** |  |
| **3.** | **Materials:** Describe any physical or informational materials used in the intervention, including those provided to participants or used in intervention delivery or in training of intervention providers. Provide information on where the materials can be accessed (e.g. online appendix, URL). | (1) Practice staff will be provided with a manual for conducting the audit and delivering the other components of the intervention  (2) Short script provided to GPs and practice nurses used in face-to-face and phone reminders.  (3) GP-endorsed letter  (4) Information leaflet  All materials will be provided to practices by study researchers. |
| **4.** | **Procedures:** Describe each of the procedures, activities, and/or processes used in the intervention, including any enabling or support activities. | The practice-level components consist of:  *Educational meeting and provision of local technical assistance**   1. Briefing (20-30 minutes) delivered at the practice by a member of the research term to outline the purpose of the intervention, benefits to practice when patients attend (e.g. timely results, access to local service), informing them patients are more likely to attend screening if someone at the practice prompts or encourages them to do so, and explaining how the intervention will be delivered after the audit is complete. The researcher will outline the estimated time each component is expected to take. All staff should attend the briefing, if feasible. The briefing may be delivered during pre-existing lunchtime continuing professional development (CPD) meetings. 2. Training (one hour) delivered at the practice by a member of the research term to person responsible for the audit. Specifically, the training will explain the audit manual including how to check and register patients with the national programme, how to anonymise data, and how to add an electronic alert to patient file. During training the researcher will also specify the following information should be logged on the audit file once phone call reminder delivered: 1. They have phoned patient (and date) 2. They got through to patient 3. They delivered scripted message.   *Audit and feedback*   1. A member of the practice staff conducts an audit of their patients with diabetes (type 1 or type 2) aged ≥ 18 years (or a sub-sample of 100 patients if > 100 patients with diabetes at the practice) to identify non-attenders and re-audit at 6 months to identify any changes in attendance. A paper-based or Excel template can be used to record data during the audit. The staff member will conduct a data search; filters will be applied to the GP software, to identify all adults with diabetes.   *Remind clinicians*   1. A practice nurse or administrator will add an electronic alert to the records of non-attenders based on the results of the audit. Prompts will be automatically programmed to appear at the start of each consultation. GPs and practice nurses will be asked to delete the alert if the intervention (i.e. brief messages, leaflet) were delivered to patients. 2. Laminated prompt script given to each GP and practice nurse to guide their delivery of the in-person and phone reminder.   *Payment schemes*   1. Practices will be given a set payment to cover the costs of conducting the practice audit and delivering the intervention components. A standard fee of €500 at study entry with further payment following study cessation based on number of patients audited (€5 per patient); up to additional €500, and on receipt of anonymised audit file.   *Intervene with patients* *to enhance uptake*  The patient-level components include :   1. In person reminder during consultation with health professional; 2. And/or phone reminder, and; 3. Follow-up GP-endorsed letter reminder on practice-headed paper, and; 4. Patient information leaflet provided to patients with persuasive key messages under ‘Why they should attend’ and instruction ‘What should they do next’. |
|  | **WHO PROVIDED** |  |
| **5.** | For each category of intervention provider (e.g. psychologist, nursing assistant), describe their expertise, background and any specific training given. | Health services researcher with a background in Public Health  Criteria for the researcher:   1. Has experience of setting up and conducting practice audits and can provide technical support if necessary. 2. Is familiar with the evidence on interventions to improve attendance at retinopathy screening. 3. Is familiar with, and has analysed data on, the barriers and enablers of attendance. 4. Can advise on acceptability or feasibility issues with the intervention and study protocol.   Criteria for clinical provider:   1. Has ongoing contact with patients at the practice (e.g. practice nurse) |
|  | **HOW** |  |
| **6.** | Describe the modes of delivery (e.g. face-to-face or by some other mechanism, such as internet or telephone) of the intervention and whether it was provided individually or in a group. | The practice staff intervention (briefing and training) will be delivered in a face to face group or one-on-one session with the researcher using a protocol. The patient intervention will be delivered 1) in person during consultations, by GPs or practice nurses; 2) by phone by practice nurses, and 3) via letter endorsed by the GP. |
|  | **WHERE** |  |
| **7.** | Describe the type(s) of location(s) where the intervention occurred, including any necessary infrastructure or relevant features. | The intervention will be delivered in general practices. The patient intervention will be embedded into existing consultations, and reminders will be delivered by GPs or nurses. |
|  | **WHEN and HOW MUCH** |  |
| **8.** | Describe the number of times the intervention was delivered and over what period of time including the number of sessions, their schedule, and their duration, intensity or dose. | The intervention will be delivered over a 6-month period. The staff intervention will be delivered once and last for approximately 20 to 30 minutes for the brief training, and one hour for the training in the audit protocol with dedicated staff member(s) i.e. practice nurse and/or administrator. The audit will be completed within one calendar month of starting the study. The patient intervention will be delivered once to patients identified as non-attenders. Patients who attend the practice during the trial period will receive the in-person reminder message together with phone call and follow up letter. Reminder phone calls will be carried out within two weeks of completing the practice audit and identifying non-attenders. Follow-up reminder letters will be sent within two to three days of the phone call. |
|  | **TAILORING** | N/A |
| **9.** | If the intervention was planned to be personalised, titrated or adapted, then describe what, why, when, and how. | There is some scope for planned adaptations. For example, practices may decide locally which staff member will be responsible for different components; i.e., conducting audit, adding prompts, delivering reminders. |
|  | **MODIFICATIONS** |  |
| **10.^ǂ^** | If the intervention was modified during the course of the study, describe the changes (what, why, when, and how). | N/A |
|  | **HOW WELL** |  |
| **11.** | Planned: If intervention adherence or fidelity was assessed, describe how and by whom, and if any strategies were used to maintain or improve fidelity, describe them. | Fidelity of intervention delivery will be assessed using practice progress-reports (e.g. logging patient phone calls), monthly follow-up phone calls to practices by a study researcher, interviews and focus groups with patients and practice staff at study cessation.  Further details are provided in the protocol paper. |
| **12.** | Actual: If intervention adherence or fidelity was assessed, describe the extent to which the intervention was delivered as planned. | N/A |

*Implementation strategies are mapped to those outlined in the taxonomy developed by Powell et al.
