# Supplementary material for: Feasibility of an implementation intervention to increase attendance at diabetic retinopathy screening: protocol for a cluster randomised pilot trial
Source: Pilot Feasibility Stud. 2020 May 12;6:64. doi: 10.1186/s40814-020-00608-y (PMC7216495; doi:10.1186/s40814-020-00608-y)
Supplement: Supplementary file 2 — Additional file 2:. Messages delivered as part of the short script. Intervention material [file 40814_2020_608_MOESM2_ESM.docx]

| Messages delivered as part of the short script |
| --- |
| - Our records show that you may not have participated in diabetes eye screening recently with the national RetinaScreen programme. Do you know about Diabetic RetinaScreen? RetinaScreen is the national diabetes retinopathy screening programme which offers free, regular retinopathy screening to people with diabetes. |
| - Would you mind if I ask why you haven’t participated? |
| - We strongly recommend that you participate in screening. |
| - Diabetes can cause damage to your eyes. This complication of diabetes is called retinopathy. *Everyone* with diabetes is at risk of developing retinopathy. Even if you have no symptoms, your eyes may already be showing signs of damage. Attending screening dramatically increases the chance of this damage being picked up early and treated in time. |
| - After the screening test most people are reassured that their eyes are fine. |
| - The routine eye checks you might have with your optician are different to the screening provided by the national screening service. Even if you are attending an optician you should still participate in the national programme. |
| - It’s a free service and it’s very easy to participate; all you need to do is ring RetinaScreen. If you want help with the next steps come in and speak with the nurse. We will be sending you a reminder letter in a day or two with some more information. |
