# Supplementary material for: Feasibility of an implementation intervention to increase attendance at diabetic retinopathy screening: protocol for a cluster randomised pilot trial
Source: Pilot Feasibility Stud. 2020 May 12;6:64. doi: 10.1186/s40814-020-00608-y (PMC7216495; doi:10.1186/s40814-020-00608-y)
Supplement: Supplementary file 3 — Additional file 3:. Information leaflet and intervention material [file 40814_2020_608_MOESM3_ESM.pdf]

Screening Could Save Your Sight

A message from your GP about diabetes eye screening

Why we think you should attend diabetes eye screening

Diabetes eye screening is provided by RetinaScreen, the free national diabetes retinopathy screening programme.

Here are 5 reasons why we think you should participate:

1

Diabetes can cause damage to the blood vessels at the back of your eye which affects your sight.
This complication of diabetes is called retinopathy.

2

Everyone with diabetes is at risk of developing retinopathy.
Diabetes can harm your eyes long before you have symptoms so it is very important that you attend screening. Diabetes eye screening is an *essential* part of your regular diabetes care.

3

Routine eye checks you might have with your optician are different to the national screening service.
Even if you attend an optician regularly you should still attend screening with the national RetinaScreen service.

4

We understand you might be worried about attending screening or finding out something is wrong, **but most people who attend screening are reassured that their eyes are fine.**

5

Attending screening dramatically increases the chance of damage being picked up early and treated in time.
Untreated diabetic retinopathy is a common cause of sight loss in people with diabetes, but it is treatable if picked up early.

Remember RetinaScreen is free

What should I do next?

Ring RetinaScreen by calling 1800 45 45 55
or email them at info@diabeticretinascreen.ie

OR

Fill out the enclosed consent form and send it to RetinaScreen using the freepost envelope provided

If you want help with the form, come into the practice and we can fill it out together

What will happen at my appointment?

You will be called into the consultation room to have drops put into your eyes. You might find these uncomfortable and your vision might become blurred, but this is temporary and the drops are not harmful. Then, the back of your eyes will be photographed with a digital camera. This camera doesn't touch your eyes at any stage. That's it. The whole process will take about half an hour.

What if the appointment date or time don't suit me?

Give RetinaScreen a quick ring and ask for a new appointment. The staff at RetinaScreen are used to this and they're happy to help. They will do everything they can to work with your schedule and find an appointment that suits you.

If you do just one thing today, ring RetinaScreen.

Diabetic RetinaScreen
An Clár Náisiúnta Scagthástála Reitiní do Dhiaibéitigh
The National Diabetic Retinal Screening Programme
